# Effect of Almond Shell Waste on Physicochemical Properties of Polyester-Based Biocomposites

**DOI:** 10.3390/polym12040835

**Published:** 2020-04-06

**Authors:** Marina Ramos, Franco Dominici, Francesca Luzi, Alfonso Jiménez, Maria Carmen Garrigós, Luigi Torre, Debora Puglia

**Affiliations:** 1 Department of Analytical Chemistry, Nutrition & Food Sciences, University of Alicante, San Vicente del Raspeig, ES-03690 Alicante, Spain; marina.ramos@ua.es (M.R.); alfjimenez@ua.es (A.J.); 2 Department of Civil and Environmental Engineering, University of Perugia, 05100 Terni, Italy; francodominici1@gmail.com (F.D.); francesca.luzi@unipg.it (F.L.); luigi.torre@unipg.it (L.T.)

**Keywords:** almond shell waste, reinforcing, polyester-based biocomposites, physicochemical properties, disintegration

## Abstract

Polyester-based biocomposites containing INZEA F2^®^ biopolymer and almond shell powder (ASP) at 10 and 25 wt % contents with and without two different compatibilizers, maleinized linseed oil and Joncryl ADR 4400^®^, were prepared by melt blending in an extruder, followed by injection molding. The effect of fine (125–250 m) and coarse (500–1000 m) milling sizes of ASP was also evaluated. An improvement in elastic modulus was observed with the addition of< both fine and coarse ASP at 25 wt %. The addition of maleinized linseed oil and Joncryl ADR 4400 produced some compatibilizing effect at low filler contents while biocomposites with a higher amount of ASP still presented some gaps at the interface by field emission scanning electron microscopy. Some decrease in thermal stability was shown which was related to the relatively low thermal stability and disintegration of the lignocellulosic filler. The added modifiers provided some enhanced thermal resistance to the final biocomposites. Thermal analysis by differential scanning calorimetry and thermogravimetric analysis suggested the presence of two different polyesters in the polymer matrix, with one of them showing full disintegration after 28 and 90 days for biocomposites containing 25 and 10 wt %, respectively, under composting conditions. The developed biocomposites have been shown to be potential polyester-based matrices for use as compostable materials at high filler contents.

## 1. Introduction

Almond is characterized by its high nutritional value, although information reported so far mainly concerns its edible kernel or meat. Other parts also present in the almond fruit are the middle shell, outer green shell cover or almond hull and a thin leathery layer known as brown skin of meat or seed coat [1]. Almonds are used as a fruit in snack foods and as ingredients in a variety of processed foods, especially in bakery and confectionery products. However, almond production generates large amounts of almond by-products since the nutritional and commercial relevance of almonds is restricted to the kernel. In particular, almond shell is the name given to the ligneous material forming the thick endocarp or husk of the almond (*Prunus amygdalus L.*) tree fruit. It is principally composed of cellulose (ranging from 29.8 to 50.7 wt %), hemicellulose (from 19.3 to 29.0 wt %) and lignin (from 20.4 to 50.7 wt %) [1]. This by-product is normally incinerated or dumped without control, which results in the production of large amounts of waste and pollution [2]. Several researchers have focused on different alternatives for using almond shell wastes based on their potential uses as biomass to produce renewable energy [3]; as a source of organic biopesticides [4], heavy metal adsorbents [5], dye adsorbents [6], growing media [7], the preparation of activated carbons [8] and xylo-oligosaccharides [9], antioxidants [10] or as additives in eco-friendly composites [11,12,13].

The development of eco-friendly composites arises from the need for reducing environmental problems generated by industrial processes. In this scenario, agricultural waste utilization has become a potential option for the development of eco-friendly composites. This powerful area of interest presents several benefits such as biodegradability in combination with bio-based or natural polymers, light weight, low cost and easy processing [14,15]. Among the wide variety of lignocellulosic wastes, almond shell powder has been already considered as filler for commodity plastics, such as polypropylene [16,17,18,19,20], polyethylene [21], poly(methyl methacrylate) [22] and toughened epoxies [23,24].

Looking at a more environmentally friendly use, the role of ASP has been recently studied in enhancing the mechanical performance of some melt compounded biopolymers [25,26,27]. Nonetheless, due to the lack of miscibility between hydrophobic polymer matrices and highly hydrophilic almond shell fillers, the obtained green composites usually presented poor ductility and low thermal stability. In order to increase the interaction between them, several solutions have been proposed, such as silanization, acetylation and maleic anhydride modification [28]. Plasticizers could also act as internal lubricants, thus allowing chain mobility, which enhances processability and improves thermal stability and ductility. Recently, vegetable oils have been proposed as environmentally friendly compatibilizers as an alternative to conventional petroleum-based ones [29]. Specifically, maleinized linseed oil (MLO) has been used as a compatibilizer in biopolymer/ASP composites [11,30,31]. In these works, authors discussed plasticization and compatibilization effects provided by MLO due to the interaction between succinic anhydride polar groups contained in MLO and hydroxyl groups in ASP (hydroxyl groups in cellulose). The compatibilizing effect was obtained by melt grafting for the formation of new carboxylic ester bonds through the reaction of maleic anhydride functionalities present in MLO with the hydroxyl groups of both the polyester terminal chains and cellulose on the ASP surface. On the other hand, the possibility of improving the stress transfer between the filler and the polymer can be realized by reactive processing with chain extenders [32].

The main aim of the present work is the development and characterization of new biocomposites prepared using a commercial INZEA^®^ biopolymer (mainly composed of a polyester-based matrix) containing almond shell powder at 10 and 25 wt % contents. The effect of adding two different milling sizes (125–250 μm and 500–1000 μm) in the biocomposites preparation was also evaluated. In addition, the potential of maleinized linseed oil as a compatibilizer was studied. The effect of this vegetable-oil-derived compatibilizer was also compared with a conventional epoxy styrene-acrylic oligomer (Joncryl ADR 4400) in terms of mechanical properties, thermal stability and blend morphology. Biocomposites containing 10 and 25 wt % of ASP at two grinding levels were submitted to a disintegration test in order to verify the effectiveness of the developed polyester/ASP composites to be used as compostable materials.

## 2. Materials and Methods

### 2.1. Materials

INZEA^®^ biopolyester commercial grade, with a density of 1.23 g cm^−3^ measured at 23 °C, a moisture content <0.5% and a melt flow rate of 19 g/10 min (2.16 kg, 190 °C), was kindly supplied by Nurel (Zaragoza, Spain). Almond shell (AS) waste used as filler was supplied by Fecoam (Murcia, Spain) as an agricultural by-product and pulverized with a high-speed rotor mill (Ultra Centrifugal Mill ZM 200, RETSCH, Haan, Germany). The obtained particles were sieved by selecting the sizes of the ground shells in the ranges of 125–250 μm as fine grain (F) and 500–1000 μm as coarse grain (C) to evaluate the effect of particle size in the composites. Two different compatibilizers were selected to improve the compatibility between the polyester-based matrix and the natural filler: a synthetic polymer chain extender with recognized efficacy as compatibilizer supplied as Joncryl ADR 4400^®^ (J44) (BASF S.A, Barcelona, Spain) and a biodegradable additive obtained by the maleinizing treatment of linseed oil, supplied as Veomer Lin by Vandeputte (Mouscron, Belgium, viscosity of 10 dPa s at 20 °C and an acid value of 105–130 mg KOH g^−1^).

### 2.2. Biocomposites Preparation

Biocomposite materials were obtained using the melt blending method by mixing the biopolymer matrix with the almond particles, obtained by grinding and sieving the almond shells as previously described and the additives according to the proportions shown in Table 1. A co-rotating twin-screw extruder, Xplore 5 & 15 Micro Compounder by DSM, was used by mixing at a rotating speed of 90 rpm for 3 min and setting a temperature profile of 190–195–200 °C in the three heating zones from feeding section to die. A Micro Injection Molding Machine 10 cc by DSM, coupled to the extruder and equipped with adequate molds, was used to produce samples for flexural tests according to the standards. An appropriate pressure/time profile was used for the injection of each type of sample, while the temperatures of the injection barrel and the molds were set, respectively, at 210 and 30 °C.

### 2.3. Almond and Biocomposites Characterization

#### 2.3.1. Field Emission Scanning Electron Microscopy

Morphological characterization of ASP was carried out using a field emission scanning electron microscope (FESEM), Supra 25 by Zeiss (Oberkochen, Germany). The surfaces and the fractures of biocomposites were analyzed with a FESEM Merlin VP Compact by ZEISS. In both cases, micrographs were taken using an accelerating voltage of 5 kV at different magnifications. Samples were previously gold-sputtered with an Automatic Sputter Coater, B7341 by Agar Scientific (Stansted, Essex, UK), operating with a vacuum atmosphere (0.1–0.005 mbar) and low current (0–50 mA) to provide electric conductivity.

#### 2.3.2. Thermal Characterization

Thermogravimetric analysis of ASP was performed with thermogravimetric analysis (TGA; (Seiko Exstar 6300, Tokyo, Japan). Approximately 5 mg of samples were heated from 30 to 600 °C at 10 °C min^−1^ under nitrogen atmosphere (flow rate 200 mL min^−1^).

Differential scanning calorimetry (DSC) tests were conducted for the determination of thermal events by using a DSC (Q1000, TA Instruments, New castle, DE, USA) under a nitrogen atmosphere (50 mL min^−1^). A 3 mg amount of samples were introduced in aluminum pans (40 µL) and they were submitted to the following thermal program: −30 °C to 250 °C at 10 °C min^−1^, with two heating and one cooling scans.

The thermal degradation behavior of biocomposites in composting conditions was evaluated by thermogravimetric analysis (TGA/SDTA851e/SF/1100, Mettler Toledo, (Schwarzenbach, Switzerland). Around 5 mg of samples were used to perform dynamic tests in a nitrogen atmosphere (200 mL min^–1^) from 30 °C to 700 °C at 10 °C min^−1^.

#### 2.3.3. Mechanical Properties

Flexural tests were carried out by using a universal test machine LR30K (Lloyd Instruments Ltd., Bognor Regis, UK) at room temperature. A minimum of five different samples was tested using a 0.5 kN load cell, setting the crosshead speed to 2 mm min^−1^ for three points bending test, as suggested by ISO 178 Standard.

#### 2.3.4. Disintegrability in Composting Conditions

Disintegration tests in composting conditions were performed, in triplicate, by following the ISO 20200 Standard method using a commercial compost with a certain amount of sawdust, rabbit food, starch, oil and urea [33]. Tested samples were obtained from the previously prepared dog-bone-shaped bars, which were cut in pieces (5 × 10 × 2 mm^3^), buried at a 5 cm depth in perforated boxes and incubated at 58 °C. The aerobic conditions were guaranteed by mixing the compost softly and by the periodical addition of water according to the standard requirements.

Different disintegration times were selected to recover samples from burial and further tested: 0, 4, 7, 15, 21, 28, 40, 69 and 90 days. Samples were immediately washed with distilled water to remove traces of compost extracted from the container and further dried at 40 °C for 24 h before gravimetric analysis. The disintegrability value for each material at different times was obtained by normalizing the sample weight with the value obtained at the initial time.

The evolution of disintegration was monitored by taking photographs of recovered samples for visual evaluation of physical alterations with disintegration time. In addition, thermal (DSC, TGA) properties upon disintegrability tests were also studied.

### 2.4. Statistical Analysis

Statistical analysis of experimental data was performed by one-way analysis of variance (ANOVA) using SPSS 15.0 (IBM, Chicago, IL, USA) and expressed as means ± standard deviation. Differences between average values were assessed based on the Tukey test at a confidence level of 95% (*p* < 0.05).

## 3. Results

### 3.1. Characterization of Almond Shell Powder

#### 3.1.1. Morphological Analysis

**Figure 1:**
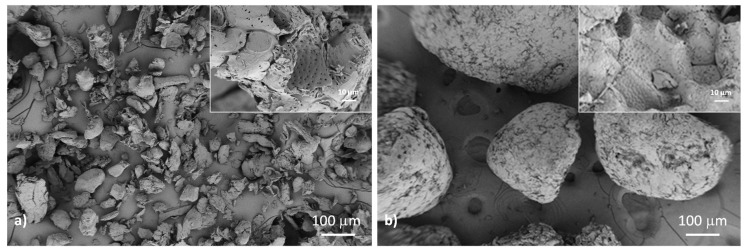
FESEM images of fine (**a**) and coarse (**b**) almond shell powders.

Low-magnification FESEM micrographs of almond shell waste at the two different studied sizes (125–250 μm, fine, and 500–1000 μm, coarse), reported in Figure 1, showed a general view typical of fillers obtained after grinding and sieving processes. Almond shell was shown to have a sheet-like structure. In addition, a series of 1 μm pores were observed on the almond surface (see inserts) [34]. Most of the particles were characterized by a spherical shape, though some aggregates, as well as flat and long rod-like particles, were also observed. A detail of the particle surface can be seen in the high-magnification FESEM images. The micrographs revealed that the particles were irregular in shape and presented a rough surface, more likely resulting from the crushing process due to the high hardness of this type of filler. Some granular features can also be observed, which resemble the original grainy and wavy structure of almond shell [35].

#### 3.1.2. Thermal Properties

**Figure 2:**
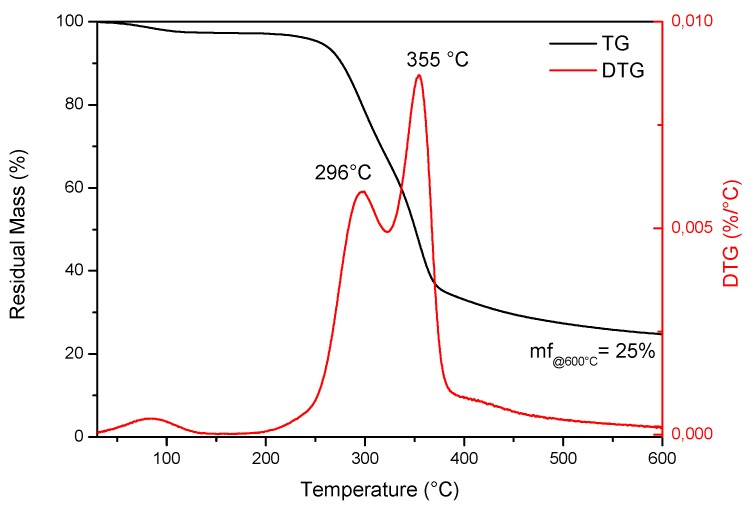
TGA analysis of almond coarse shell powder.

TG and derivative DTG profiles obtained for ASP (coarse size) under the nitrogen atmosphere at a heating rate of 10 °C min^−1^, reported in Figure 2, showed the typical thermal degradation profile for biomasses with three well-demarked steps for moisture release, devolatilization and char formation. Weight loss in the lower temperature region can be attributed to the loss of moisture, while major weight loss was observed at temperatures ranging from 225 to 365 °C, over which hemicellulose and cellulose decomposition occurs, leading to the formation of pyrolysis products (volatiles, gases and primary biochar) [36]. This phenomenon was followed by a slow weight loss until 600 °C, which was attributable to the continuous devolatilization of biochar caused by a further breakdown of C–C and C–H bonds.

### 3.2. Characterization of ASP Biocomposites

#### 3.2.1. Flexural Tests

**Figure 3:**
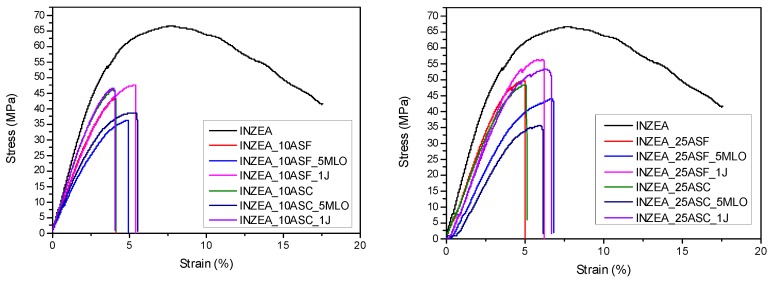
Stress–strain curves of polyester-based biocomposites containing 10 and 25 wt % of ASP at two grinding levels (F, C), with or without compatibilizers.

Flexural tests provided information on the effect of the amount and size of ASP incorporated in the composite, as well as on the effect of adding the studied compatibilizing additives. In general, results show that the addition of the filler did not improve the maximum strength or strain at break with respect to the reference polymer matrix (Table 2). The elastic modulus of the biocomposites was improved only in formulations containing 25 wt % of both fine and coarse filler. The comparison of flexural tests for formulations with 10 wt % of ASP (Table 2 and Figure 3) showed that the size of the coarse grain gives greater rigidity than the fine grain by increasing strength and modulus but slightly reducing elongation. In fact, the presence of 10 wt % of fine filler in the INZEA_10ASF biocomposite produced a maximum strength value of 44 MPa with a flexural modulus of 1473 MPa, lower than the biocomposite with the coarse filler INZEA_10ASC, which showed σ_max_ = 47 MPa and E = 1699 MPa values. Nabinejad et al. [37] reported that the surface roughness and high surface area of coarse powder particles could have a positive effect on the mechanical performance of composites, being able to restrict the polymer chain mobility. Porosity and roughness of the hydrophilic surface for coarse almond shell powder would be expected to increase its wettability by the polymer matrix. So, as a result, the INZEA_10ASC composite showed high stiffness values compared to composites containing fine filler (INZEA_10ASF) with low surface roughness and porosity. Additionally, results from Zaini et al [38] confirmed that composites filled with a larger-sized filler showed higher modulus, tensile and impact strengths, particularly at high filler loadings.

The addition of 5 wt % of MLO in the biocomposite formulation produced a compatibilizing effect lower than expected. In fact, the improvement in deformability moved from 4.4% to 4.9% of INZEA_10ASF_5MLO and from 4.0% to 5.4% of INZEA_10ASC_5MLO in the formulations with MLO and 10 wt % ASP fine and coarse, respectively, with a consistent reduction in both flexural strength and flexural modulus. If the modest plasticizing effect is excluded, the MLO did not produce improvements of the interface strength between the natural filler and the biopolymer matrix. Effectiveness of maleinized linseed oil has been demonstrated in composites based on lignin fillers and, specifically, from ground almond shells and some biodegradable polymers, such as poly(lactic acid) (PLA) and lignin. In this case, the poor result obtained with the INZEA matrix should be attributed to the particular composition of the commercial biopolymer used (a bio-based blend mainly composed of a polyester matrix) [30,39,40]. Joncryl ADR 4400 added to the formulation with fine particles INZEA_10ASF_1J produced an increase in flexural strength from 44 to 48 MPa. This improvement is due to the compatibilizing effect of the polymer chain extender, which improves the adhesion between the matrix and the particles allowing a greater deflection. This effect is highlighted by the perfect overlap between the σ−ε curve of the unmodified INZEA_10ASF and the INZEA_10ASF_1J curve which, thanks to the J44, extends up to 5.5% increasing the flexural strength [41]. The effect of Joncryl was negligible on biocomposites with coarse grain, since the compatibilization effect at the interface between the matrix and the filler was much lower (about 6%) than biocomposites with fine particles. In fact, when simplifying the particles as spherical and calculating the ratios between coarse and fine surfaces, with the same wt % content, an area ratio of 0.0625 was obtained.

When increasing the quantity of filler to 25 wt %, an increase in the rigidity of the biocomposites was obtained. The result is a general increase in flexural strength and moduli, which corresponds to a reduction in deflection. Even in formulations with a higher content of the natural filler, MLO did not produce any other effects than those already shown in the set with 10 wt % of ASP. The flexural strength of the biocomposite containing 25 wt % of fine particle (INZEA_25ASF) rises to 50 MPa (44 MPa for INZEA_10ASF), while the INZEA_25ASC composite maintains the same value of 47 MPa as 10 wt % of filler, highlighting the achievement of the plateau of the coarse grain reinforcement. In the case of the higher amount of ASP, the compatibilizing effect of Joncryl appears even more evident, since a better interface bonding between the matrix and the filler was achieved. INZEA_25ASF_1J showed a further increase in flexural strength reaching 56 MPa, with an improvement in modulus to 2555 MPa and without excessively reducing the flexural deflection. In addition, INZEA_25ASC_1J with Joncryl showed an improvement in strength going up from 47 to 53 MPa, but in this case, the compatibilizing effect of J44 was less evident, because it occurred on a smaller interface surface, compared to fine-grain-sized biocomposites, due to the larger particle size [13].

#### 3.2.2. Morphological and Thermal Analysis

**Figure 4:**
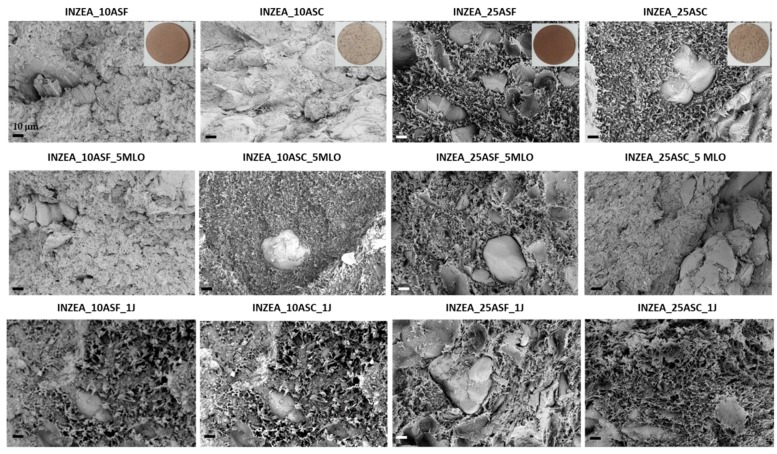
FESEM images of INZEA-based biocomposites with 10 and 25 wt % of ASP at the two grinding levels (fine, coarse), with or without compatibilizers.

In Figure 4, FESEM images of fractured surfaces for INZEA/ASP composites (uncompatibilized and compatibilized biocomposites) are reported. As it can be observed, in the case of the unmodified matrix, the polymer‒particle adhesion was very poor, both at low and high ASP contents, so important gaps can be found between the particles and the surrounding polyester matrix [11]. This morphological observation correlates with the above-described mechanical performance of the unmodified INZEA composites, in which the presence of ASP did not contribute to an improvement in mechanical performance. The addition of 5 wt % MLO provides some interaction as the gap seems to be reduced, indicating a limited but good compatibilizing effect of MLO modifier, but at the same time, the presence of microsized voids in the matrix compatibilized with 5 wt % of MLO was visible [30]. In the presence of a styrene-acrylic-based compatibilizer and oligomeric agent, an improvement in the interface with ASF and ASC was noted at higher contents (25 wt %), even if biocomposites containing the higher amount presented some gaps at the interface. So, it can be concluded the chain extender was effective at both filler contents [42].

**Figure 5:**
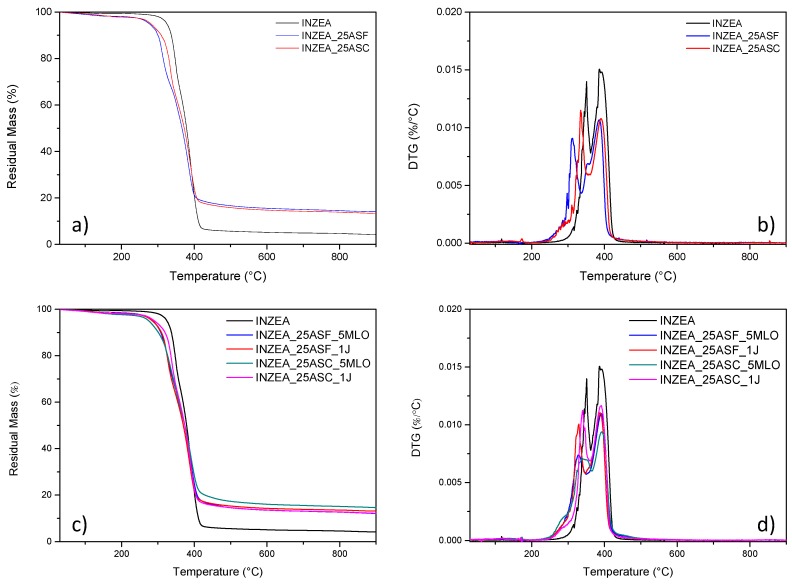
TG/DTG curves of INZEA-based biocomposites with 25 wt % of ASP at the two grinding levels (**a**,**b**) and INZEA biocomposites with 25 wt % of ASP in the presence of MLO or Joncryl compatibilizers (**c**,**d**).

Figure 5a,b shows the TG/DTG thermograms of the INZEA matrix with the addition of 25 wt % ASP at the two different grinding sizes. Only the trends in thermal behavior for the biocomposites containing the higher ASP content have been reported, being the ones at 10 wt % essentially inline (data not shown). The presence of a double degradation peak for the INZEA neat matrix gives us an indication of a material that degrades in two steps around 350 and 400 °C, that could match with the possible degradation temperatures of PLA and poly(butylene succinate) (PBS) polyesters. It is also important to note that at 900 °C, even the unmodified INZEA matrix maintains a residual mass of ca. 5 wt %, which is increased by the presence of the fillers. This is in accordance with the possible presence of an inorganic filler in the formulation of the commercial product.

The TG curves corresponding to INZEA/ASP composites showed an evident decrease at the onset degradation temperature from 324 °C for neat INZEA to 275 °C and 280 °C, respectively, for INZEA_25ASC and INZEA_25ASF (Table 3). This behavior is essentially due to the relatively low thermal stability of the lignocellulosic filler, which initiated its degradation at 198 °C (Figure 2), and negatively contributed to the reduction of the global thermal stability of the INZEA-based biocomposites, in agreement with previous studies on the same bio-based reinforcement [17,20]. The introduction of the ASP filler mainly affected the thermal stability of the polyester component with the lower T_peak_ temperature (maximum mass loss rate): in detail, T_peak1_ moved from 351 °C to 311 °C and 334 °C for INZEA_25ASF and INZEA_25ASC, respectively, while the temperature for the second peak (T_peak2_) remained practically unchanged for all the different composites (Table 3). This behavior was related to the higher amount of ASP incorporated and its degradation over this temperature range. According to other authors, cellulose, hemicelluloses and lignin show a broad temperature range starting at about 250 °C and ending at 450 °C in a progressive weight loss process, and this degradation can reduce the thermal stability of biopolyesters [43,44,45]. Similar findings were reported by Liminana et al. [31] who observed a decrease of 11.2 °C in thermal stability of PBS with the addition of 30 wt % of almond shells.

The addition of MLO (Figure 5c,d) exerted a limited positive effect on the overall thermal stability of the biocomposites (Table 3). Specifically, the onset degradation temperature increased to 285 °C for INZEA_25ASF_5MLO and substantially remained unchanged for INZEA_25ASC_5MLO (if compared to uncompatibilized matrices). On the other hand, the temperature of T_peak1_ was significantly improved, in comparison with the unmodified ASP biocomposites, at the two grinding sizes. In particular, T_peak1_ was delayed up to 328 °C for the INZEA_25ASF_5MLO material and 341 °C for the INZEA_25ASC_5MLO material. This increase in thermal stability could be directly related to the chemical interaction achieved by MLO, due to the establishment of covalent bonds between the lignocellulosic fillers and the polyester matrix. In addition, MLO could also provide a physical barrier that obstructs the removal of volatile products produced during decomposition. A similar effect on thermal stability was recently reported for PLA and epoxidized palm oil blends [46].

It has been also reported that the reactive extrusion of aliphatic polyesters, such as PLA, with styrene-epoxy acrylic oligomers can provide an increase in thermal stability due to the branching effect obtained during the extrusion process. As it has been found in our case (Figure 5c,d), the presence of Joncryl provided enhanced thermal resistance for the low T_peak_ polyester phase, moving this value from 311 °C and 334 °C (INZEA_25ASF and INZEA_25ASC), respectively, to 329 °C and 339 °C for INZEA_25ASF_1J and INZEA_25ASC_1J (Table 3). This effect was already observed by Lascano et al in poly(lactic acid)/poly(butylene succinate-co-adipate) blends containing Joncryl [47].

### 3.3. Disintegration Tests

According to the characterization results previously obtained, the disintegrability of polyester-based biocomposites with 10 and 25 wt % of ASP at two grinding levels (F, C) was studied to evaluate their degradation in natural environments. Formulations including the two studied compatibilizing agents were not included in this study, since the main goal of this characterization was the analysis of ASP size and content on disintegration behavior of the reference matrix.

**Figure 6:**
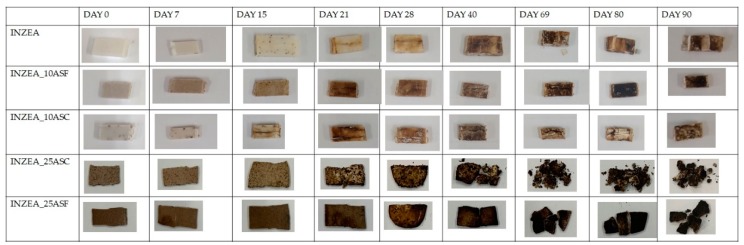
Visual appearance of INZEA-based biocomposites with 10 and 25 wt % of ASP at two grinding levels (F, C) at different testing days at 58 °C.

The visual evaluation of all samples at different degradation times was carried out and results are shown in Figure 6. Some changes in sample surfaces submitted to composting conditions were clearly appreciable, showing all samples considerable modifications in color and morphology after 15 treatment days. These modifications can be associated to the beginning of the polymer matrix degradation, which can be related to the direct contact of biocomposites with the compost, by a gradually microorganism erosion from the surface to the bulk and to the moisture absorption, as it has been reported in the literature [45]. After 69 days of study, samples with 25 wt % of ASP have completely lost their morphology, and small fragments can be observed (in particular for INZEA_25ASC). In addition, differences in color could be observed between samples with different amounts of ASP. Formulations with 10 wt % of ASP showed disintegration behavior similar to that of INZEA neat during all the study.

**Figure 7:**
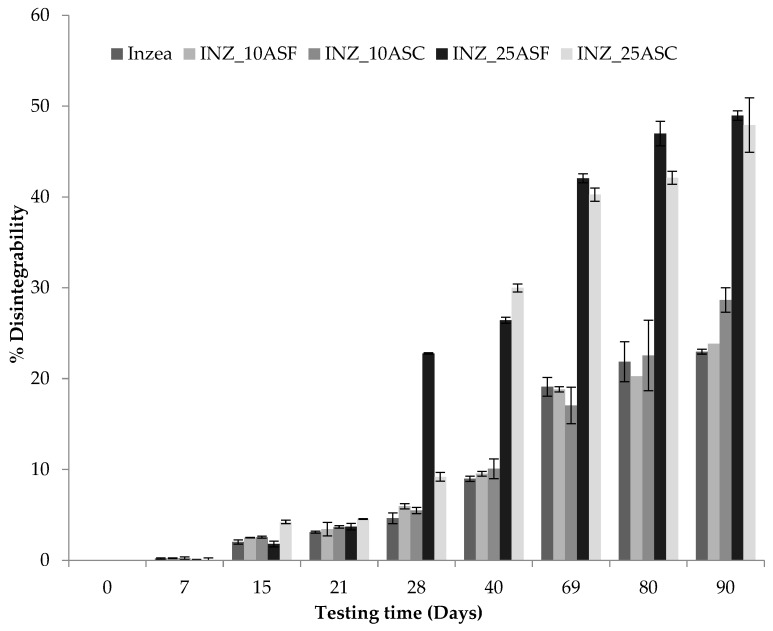
Disintegrability (%) of INZEA-based biocomposites with 10 and 25 wt % of ASP at two grinding levels (F, C) as a function of degradation time under composting conditions at 58 °C (mean ± SD, *n* = 3).

Figure 7 shows the evolution of disintegrability values (%) as a function of testing time for all biomaterials. According to de Olivera et al. [48], the first stage of the biodegradation mechanism is the release of enzymes that can cause the hydrolysis of the polymer matrix and break of polymer chains, creating functional groups capable of improving hydrophilicity and the microorganism’s adhesion on the surface of the polymer matrix. The results obtained at longer times suggested that physical degradation progressed slowly with burial time, indicating that microorganisms required more time to produce suitable enzymes capable to break down polymer chains, resulting in an incomplete loss of the initial morphology and general rupture after 90 days for formulations with 10 wt % of ASP and the INZEA control. Formulations with 25 wt % of ASP significantly increased their disintegrability ratio compared to formulations with 10 wt % of ASP and INZEA control after 28 days of study, probably due to the higher amount of ASP, which enhances the high biodegradability of lignocellulosic residues, and to the poor fiber/matrix adhesion allowing and facilitating microorganisms attack and biodegradation rate by promoting biofouling and the adhesion of microorganisms to the surface [49]. Moreover, this increase in the disintegrability rate of the polymer matrix could be due to the presence of hydroxyl groups in ASP [49], which could play a catalytic role on the hydrolysis of the polymer, inducing an acceleration of polymer weight loss due to the higher filler addition [50]. Similar results were found by Wu [51] and de Oliveira et al. [48] when studying the biodegradation of composites obtained with poly(butylene adipate-co-terephthalate) and different natural fillers. The authors observed that the biodegradation rate of the composites increased with filler content. In this sense, the presence of high amounts of ASP can be related to a greater discontinuity in the polymer matrix which could facilitate water penetration into the biocomposites producing a huge modification of the surface and generating a natural environment conducive to the growth of microorganisms [48]. After 90 days of study, almost 50% of the materials were disintegrated under composting conditions. However, lower weight loss ratios were obtained with lower amounts of ASP. This behavior suggests that the disintegration rate is more influenced and dependent on the polymer matrix being slower at lower ASP contents.

**Figure 8:**
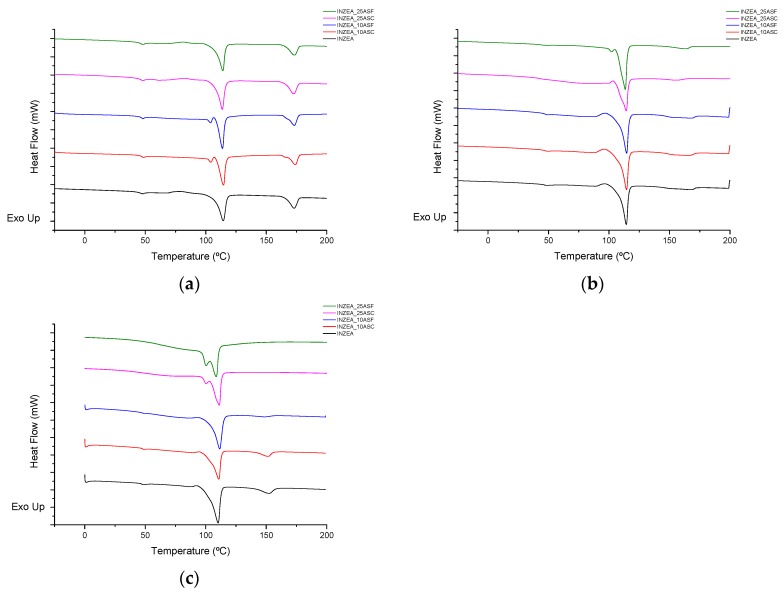
DSC thermograms of INZEA-based biocomposites with 10 and 25 wt % of ASP at two grinding levels (F, C) after different degradation times ((**a**): 0 days, (**b**): 28 days, (**c**): 90 days) at 58 °C during the first heating scan (10 °C min^−1^).

Figure 8 shows the DSC thermograms obtained during the second heating scan for all formulations as a function of composting time (0, 28 and 90 days). Two different peaks were observed at day 0, around 110 and 170 °C, indicating the presence of two main polyesters in the polymer matrix, in agreement with the behavior previously observed by TGA (Section 3.2.2). A similar DSC profile for the first and second peaks was described by Liminana et al. and Quiles-Carrillo et al. for the characterization of PBS-based and PLA-based composites reinforced with similar amounts of almond shells, respectively [12,52]. Both melting peak temperatures remained practically invariable after the addition of ASP at the two studied contents, 10 and 25 wt % (Table 4), showing a slight modification which could be related to the formation of more perfect crystals into the polymer matrix. Similar behavior was observed by Quiles-Carrillo et al. for PLA-based composites with 25 wt % of almond shell [12]. A slight decrease in the melting enthalpy of the first peak was observed with the addition of ASP which was more pronounced at 25 wt % This effect could be related to the nucleating effect exerted by the lignocellulosic filler on semicrystalline polymers acting the cellulose crystals of the almond shells as nucleating points [11].

Regarding the disintegration study, the DSC melting temperature of the second endothermic peak moved from 168.4 ± 3.7 °C for INZEA at day 0 to 142.4 ± 0.8 °C at day 90 (Table 4). Formulations with ASP did not show significant differences at day 0 compared to the values obtained for INZEA control. This decrease in around 26 °C was related to a rapid molecular mass reduction, implying that small and imperfect crystals disappeared with degradation time [53,54]. INZEA and formulations with 10 wt % of ASP did not show significant differences in DSC values at the same disintegration time, maintaining similar values throughout the whole study. In this sense, the addition of 10 wt % of ASP could not be enough to achieve an acceptable weight loss ratio into the disintegration process under composting conditions.

Under composting conditions, a different behavior was observed for formulations with 25 wt % of ASP versus time, as the second DSC peak initially appearing around 170 °C started to disappear after 28 days of study (Figure 8). After 90 days, the appearance of the thermogram suggested that this polyester-based polymer was totally disintegrated by disappearing the corresponding glass transition temperature (around 50 °C) and melting peak around 170 °C (Table 4). This result was also related to the final appearance and percentage of disintegrability achieved in samples after 90 days. On the other hand, the observed peak around 110 °C remained unchanged after 90 days of study, with slight modifications on its profile, indicating that this polymer was not degraded yet.

This behavior was also confirmed by TGA analysis (Table 4). The obtained results demonstrated that after 28 days of study the maximum degradation temperature of the first polymer peak, T_peak1_, decreased around 70 °C respect to day 0, disappearing this degradation peak from the TGA curve after 40 days under composting conditions. These results confirm those obtained by DSC where some modification of the thermal profile was observed due to the disintegration of one main polyester component of the polymer matrix.

## 4. Conclusions

In this work, biocomposite materials were obtained based on a polyester matrix (INZEAF2) and almond shell as a reinforcing agent at 10 and 25 wt % and two different milling sizes (125–250 μm and 500–1000 μm). MLO and Joncryl ADR 4400 were studied as compatibilizers. The reinforced effect of the addition of ASP was shown by increasing the elastic modulus of the biocomposites with both fine and coarse ASP at 25 wt % The addition of MLO and Joncryl produced some compatibilizing effect at 10 wt % while some gaps at the interface, visible by FESEM at 25 wt %, did not substantially affect the overall flexural properties. A double degradation pattern was obtained by TGA for the INZEA neat matrix indicating the degradation of the material in two steps and the presence of two different polyesters in the matrix. Some decrease in thermal stability of biocomposites was shown which was more pronounced at high ASP contents and it was related to the relatively low thermal stability and disintegration of the lignocellulosic filler over the studied temperature range. Some limited positive enhancement in thermal stability was obtained by adding the studied modifiers. The addition of 10 wt % of ASP was not enough to achieve an acceptable weight loss ratio of disintegration under composting conditions, whereas around 50% of disintegration (nearly double of the polymer control) was obtained by adding 25 wt % of ASP after 90 days. At these conditions, one of the polyester-based polymers was totally disintegrated by disappearing the corresponding glass transition temperature (around 50 °C), melting peak (around 170 °C) and degradation temperature (around 350 °C). On the other hand, the second polymer with T_m_ around 110 °C was not degraded yet.

The developed composites represent an interesting approach to reduce the overall cost of bio-based polyesters increasing also the added-value potential of almond agricultural wastes, obtaining environmentally friendly materials with specific reinforced and aesthetic functionalities and contributing to the circular economy approach. Further work will be needed in order to evaluate a possible improvement in filler–matrix adhesion and disintegration rate of the biocomposites by using different compatibilizers and adding higher amounts of lignocellulosic filler without compromising the final mechanical and thermal properties.

## Figures and Tables

**Table 1:** Formulations obtained in this work and their codification.

Formulation	Biopolymer (wt %)	Milled ASP (wt %)	Grain Size *	J44 (wt %)	MLO (wt %)
INZEA	100.00				
INZ_10ASF	90.00	10	F		
INZ_10ASC	90.00	10	C		
INZ_25ASF	75.00	25	F		
INZ_25ASC	75.00	25	C		
INZ_10ASF_1J	89.10	10	F	0.90	
INZ_10ASC_1J	89.10	10	C	0.90	
INZ_25ASF_1J	74.25	25	F	0.75	
INZ_25ASC_1J	74.25	25	C	0.75	
INZ_10ASF_5MLO	85.50	10	F		4.50
INZ_10ASC_5MLO	85.50	10	C		4.50
INZ_25ASF_5MLO	71.25	25	F		3.75
INZ_25ASC_5MLO	71.25	25	C		3.75

* Fine grain (F): 125–250 µm; coarse grain (C): 500–1000 µm. J44: Joncryl ADR 4400; MLO: maleinized linseed oil.

**Table 2:** Flexural parameters for almond shell powder (ASP)-based biocomposites (mean ± SD, *n* = 5).

Formulation	σ_max_ (MPa)	ε(%) at σ_max_	E (MPa)
INZEA	66 ± 2	7.9 ± 0.2	1913 ± 13
INZEA_10ASF	44 ± 2	4.4 ± 0.4	1473 ± 16
INZEA_10ASF_5MLO	36 ± 1	4.9 ± 0.3	1170 ± 15
INZEA_10ASF_1J	48 ± 2	5.5 ± 0.6	1414 ± 8
INZEA_25ASF	50 ± 1	3.0 ± 0.2	2537 ± 35
INZEA_25ASF_5MLO	44 ± 1	4.0 ± 0.2	1838 ± 22
INZEA_25ASF_1J	56 ± 2	3.5 ± 0.1	2555 ± 61
INZEA_10ASC	47 ± 2	4.0 ± 0.3	1699 ± 32
INZEA_10ASC_5MLO	38 ± 2	5.4 ± 0.6	1300 ± 33
INZEA_10ASC_1J	47 ± 2	4.2 ± 0.4	1653 ± 24
INZEA_25ASC	47 ± 2	3.0 ± 0.2	2392 ± 77
INZEA_25ASC _5MLO	35 ± 1	3.5 ± 0.1	1732 ± 7
INZEA_25ASC _1J	53 ± 1	3.7 ± 0.2	2394 ± 25

σ_max_: flexural strength; ε at σ_max_: strain at maximum stress; E: Young’s Modulus.

**Table 3:** T_onset_ and T_peak_ values of INZEA-based biocomposites with 25 wt % of ASP at the two grinding levels with and without MLO or Joncryl compatibilizers (mean ± SD, *n* = 3).

Formulation	T_onset_ (°C)	T_peak1_ (°C)	T_peak2_ (°C)
INZEA	324 ± 1	351 ± 2	385 ± 1
INZEA_25ASF	280 ± 2	311 ± 2	391 ± 2
INZEA_25ASF_5MLO	285 ± 2	328 ± 2	388 ± 3
INZEA_25ASF_1J	285 ± 1	329 ± 1	390 ± 2
INZEA_25ASC	275 ± 2	334 ± 1	390 ± 2
INZEA_25ASC _5MLO	275 ± 3	341 ± 3	392 ± 4
INZEA_25ASC _1J	291 ± 3	339 ± 2	390 ± 3

T_onset_: initial degradation temperature; T_peak1_ and T_peak2_: first and second maximum degradation temperatures, respectively.

**Table 4:** Thermal parameters of INZEA-based biocomposites with 10 and 25 wt % of ASP at two grinding levels (F, C) after different degradation times at 58 °C (mean ± SD, *n* = 3).

Day		TGA		DSC (2^nd^ Heating)				
		T_peak1_ (°C)	T_peak2_ (°C)	T_m1_ (°C) *	ΔH_m1_ (J/g) *	T_m2_ (°C) **	ΔH_m2_ (J/g) **	T_g_ (°C)
DAY 0	INZEA	353.7 ± 4.0 ^ab^	390.7 ± 1.5 ^a^	112.3 ± 0.5 ^a^	35.0 ± 0.9 ^a^	168.4 ± 3.7 ^a^	9.3 ± 1.8 ^a^	47.2 ± 0.6 ^a^
	INZEA_10ASF	355.7 ± 1.2 ^a^	392.7 ± 0.6 ^a^	113.6 ± 0.3 ^b^	30.3 ± 0.5 ^bc^	170.2 ± 0.2 ^a^	12.6 ± 0.2 ^bc^	45.3 ± 0.1 ^a^
	INZEA_10ASC	339.9 ± 6.0 ^cd^	381.7 ± 11.3 ^a^	113.3 ± 0.1 ^b^	33.6 ± 1.5 ^ab^	170.2 ± 0.1 ^a^	13.5 ± 0.5 ^c^	45.2 ± 0.6 ^a^
	INZEA_25ASF	331.0 ± 2.0 ^d^	388.7 ± 0.6 ^a^	113.7 ± 0.2 ^b^	25.6 ± 1.7 ^d^	169.8 ± 0.2 ^a^	10.7 ± 0.2 ^ab^	45.8 ± 0.3 ^a^
	INZEA_25ASC	345.0 ± 1.0 ^bc^	393.3 ± 1.2 ^a^	114.0 ± 0.1 ^b^	28.9 ± 2.0 ^dc^	170.2 ± 0.2 ^a^	11.3 ± 1.0 ^abc^	45.9 ± 0.4 ^a^
DAY 15	INZEA	290.7 ± 1.5 ^ab^	394.3 ± 2.1 ^a^	113.6 ± 0.3 ^a^	37.5 ± 1.1 ^a^	165.9 ± 0.7 ^a^	10.0 ± 0.3 ^a^	50.0 ± 0.2 ^a^
	INZEA_10ASF	297.5 ± 5.2 ^b^	393.3 ± 0.7 ^a^	113.7 ± 0.2 ^a^	35.6 ± 0.2 ^ab^	164.3 ± 2.0 ^a^	9.8 ± 1.2 ^a^	45.2 ± 0.2 ^a^
	INZEA_10ASC	293.3 ± 1.5 ^ab^	394.0 ± 1.0 ^a^	113.7 ± 0.2 ^a^	38.2 ± 1.1 ^a^	166.5 ± 0.2 ^a^	10.3 ± 0.5 ^a^	44.9 ± 0.3 ^a^
	INZEA_25ASF	306.0 ± 5.6 ^c^	388.7 ± 0.6 ^b^	113.3 ± 0.1 ^a^	29.6 ± 0.3 ^c^	159.9 ± 0.9 ^ab^	5.5 ± 0.7 ^b^	45.4 ± 0.2 ^a^
	INZEA_25ASC	286.0 ± 1.0 ^a^	391.7 ± 1.5 ^ab^	112.7 ± 0.1 ^b^	31.3 ± 3.6 ^bc^	150.0 ± 0.4 ^c^	4.5 ± 0.5 ^b^	45.4 ± 0.2 ^a^
DAY 28	INZEA	300.3 ± 0.6 ^a^	393.3 ± 0.6 ^a^	113.6 ± 0.1 ^a^	41.4 ± 0.8 ^a^	166.1 ± 0.6 ^a^	5.2 ± 3.3 ^a^	45.2 ± 0.2 ^a^
	INZEA_10ASF	287.3 ± 1.2 ^b^	391.8 ± 1.3 ^ab^	113.7 ± 0.2 ^a^	37.6 ± 0.4 ^a^	166.2 ± 0.4 ^a^	4.5 ± 0.6 ^a^	45.5 ± 0.3 ^ab^
	INZEA_10ASC	299.7 ± 1.2 ^a^	393.3 ± 0.6 ^a^	113.8 ± 0.3 ^a^	39.9 ± 0.6 ^a^	166.3 ± 0.8 ^a^	6.2 ± 2.4 ^a^	45.5 ± 0.3 ^ab^
	INZEA_25ASF	271.3 ± 5.8 ^c^	394.0 ± 1.0 ^a^	110.9 ± 0.7 ^b^	50.2 ± 2.1 ^b^	n.d.	n.d.	44.9 ± 0.2 ^a^
	INZEA_25ASC	275.3 ± 2.1^c^	389.7 ± 0.6 ^b^	112.0 ± 0.2 ^c^	40.0 ± 4.0 ^a^	n.d.	n.d.	45.9 ± 0.2 ^b^
DAY 40	INZEA	298.0 ± 3.6 ^a^	394.3 ± 2.1 ^a^	113.6 ± 0.2^a^	44.1 ± 0.1 ^a^	166.6 ± 0.2 ^a^	13.4 ± 0.6 ^a^	44.3 ± 0.2 ^a^
	INZEA_10ASF	287.3 ± 3.2 ^b^	393.3 ± 0.6 ^a^	113.2 ± 0.1 ^ab^	41.1 ± 0.3 ^a^	167.0 ± 2.8 ^a^	4.5 ± 2.9 ^b^	46.6 ± 0.1 ^b^
	INZEA_10ASC	287.7 ± 3.2 ^b^	392.7 ± 0.6 ^a^	113.3 ± 0.1 ^ab^	40.9 ± 2.9 ^a^	166.9 ± 2.6 ^a^	5.5 ± 1.0 ^b^	45.0 ± 0.1 ^c^
	INZEA_25ASF	n.d.	382.7 ± 1.2 ^b^	112.9 ± 0.3 ^b^	38.7 ± 1.1 ^a^	n.d.	n.d.	n.d.
	INZEA_25ASC	n.d.	389.0 ± 1.0 ^c^	112.3 ± 0.3 ^c^	44.6 ± 5.3 ^a^	n.d.	n.d.	n.d.
DAY 69	INZEA	292.3 ± 6.7 ^a^	392.7 ± 2.1 ^a^	111.5 ± 0.4 ^ab^	47.4 ± 3.0 ^a^	144.8 ± 2.1 ^a^	4.1 ± 1.3 ^a^	44.8 ± 0.3 ^a^
	INZEA_10ASF	286.3 ± 2.5 ^a^	392.3 ± 1.5 ^a^	110.9 ± 0.3 ^a^	42.0 ± 0.8 ^a^	151.7 ± 4.7 ^a^	3.9 ± 0.6 ^a^	44.5 ± 0.3 ^a^
	INZEA_10ASC	283.3 ± 3.8 ^a^	391.0 ± 1.7 ^a^	111.7 ± 0.7 ^a^	50.0 ± 7.2 ^a^	144.9 ± 2.6 ^a^	4.0 ± 0.6 ^a^	45.0 ± 0.2 ^a^
	INZEA_25ASF	n.d.	382.3 ± 0.6 ^b^	111.1 ± 0.1 ^ab^	39.5 ± 0.4 ^a^	n.d.	n.d.	n.d.
	INZEA_25ASC	n.d.	391.7 ± 1.5 ^a^	112.6 ± 0.1 ^b^	45.6 ± 7.4 ^a^	n.d.	n.d.	n.d.
DAY 90	INZEA	284.7 ± 4.7 ^a^	392.3 ± 1.2 ^a^	108.9 ± 0.9 ^a^	46.0 ± 0.8 ^a^	142.4 ± 0.8 ^a^	5.4 ± 0.3 ^a^	44.8 ± 0.1 ^a^
	INZEA_10ASF	281.7 ± 1.2 ^a^	388.7 ± 0.6 ^a^	110.6 ± 0.5 ^ab^	42.8 ± 4.4 ^ab^	n.d.	n.d.	n.d.
	INZEA_10ASC	276.7 ± 3.2 ^a^	392.7 ± 0.6 ^a^	109.7 ± 1.5 ^ab^	35. ± 3.4 ^bc^	n.d.	n.d.	n.d.
	INZEA_25ASF	n.d.	374.3 ± 0.6 ^b^	110.6 ± 0.3 ^ab^	32.0 ± 2.5 ^c^	n.d.	n.d.	n.d.
	INZEA_25ASC	n.d.	384.3 ± 6.4 ^a^	111.8 ± 0.3 ^b^	40.9 ± 5.6 ^abc^	n.d.	n.d.	n.d.

DSC: * and ** correspond to the first and second melting peaks appearing in Figure 8, respectively. Different superscripts (a, b, c, ab, abc) within the same day of study indicate statistically significant different values (*p* < 0.05). T_peak1_ and T_peak2_: first and second maximum degradation temperatures, respectively; T_m1_ and T_m2_: first and second melting temperatures, respectively; ΔH_m1_ and ΔH_m2_: First and second enthalpies of fusion, respectively; T_g_: glass transition temperature.
